# Dietary Fat and Betaine Supplements Offered to Lactating Cows Affect Dry Matter Intake, Milk Production and Body Temperature Responses to an Acute Heat Challenge

**DOI:** 10.3390/ani11113110

**Published:** 2021-10-30

**Authors:** S. Richard O. Williams, Tori C. Milner, Josie B. Garner, Peter J. Moate, Joe L. Jacobs, Murray C. Hannah, William J. Wales, Leah C. Marett

**Affiliations:** 1 Agriculture Victoria Research, Ellinbank, VIC 3821, Australia; tori.c.summers@outlook.com (T.C.M.); josie.garner@agriculture.vic.gov.au (J.B.G.); peter.moate@agriculture.vic.gov.au (P.J.M.); joe.jacobs@agriculture.vic.gov.au (J.L.J.); murray.hannah@agriculture.vic.gov.au (M.C.H.); bill.wales@agriculture.vic.gov.au (W.J.W.); leah.marett@agriculture.vic.gov.au (L.C.M.); 2 Centre for Agricultural Innovation, School of Agriculture and Food, Faculty of Veterinary and Agricultural Sciences, The University of Melbourne, Melbourne, VIC 3010, Australia

**Keywords:** cattle, heat challenge, canola oil, controlled-environment chambers

## Abstract

**Simple Summary:**

Hot weather is associated with reduced milk yield of dairy cows. Supplementing the diet of lactating cows with ingredients that increase dietary energy density or that reduce internal heat production, may reduce some of the negative impacts of hot weather on milk yield. We used controlled-climate chambers to simulate a short hot-weather event and measured changes in milk yield, feed intake, and body temperature of cows fed either a fat supplement, betaine or a combination of both. Feeding cows fat resulted in improved milk production but also increased body temperature and caused a decrease in feed intake. Feeding betaine did not affect milk yield but did reduce cow body temperature at times. Contrary to our expectations, the combination of fat and betaine supplements did not result in a clear benefit in terms of milk production or body temperature. Further work is warranted to understand the interactions between dietary fat type and betaine supplements when offered to cows during periods of hot weather.

**Abstract:**

Supplementing the diet of lactating cows with ingredients that increase energy density, or reduce internal heat production, may reduce some of the negative impacts of hot weather on milk yield. Thirty-two dairy cows were assigned either: (1) basal diet only, (2) basal diet plus canola oil, (3) basal diet plus betaine, or (4) basal diet plus canola oil and betaine. The basal diet was lucerne hay, pasture silage, and grain. Cows were exposed to a four-day heat challenge (temperature-humidity index 74 to 84) in controlled-environment chambers. Canola oil supplementation increased milk production (22.0 vs. 18.7 kg/d) across all periods of our experiment and increased body temperature (39.6 vs. 39.0 °C) during the heat challenge. Betaine supplementation reduced maximum body temperature during the pre-challenge period (39.2 vs. 39.6 °C) but not during the heat challenge (40.3 °C). Cows fed canola oil had greater declines in dry matter intake (5.4 vs 2.7 kg DM) and energy corrected milk (1.3 vs. 1.0 kg) from the pre-challenge to the heat challenge than other cows. Contrary to our expectations, the combination of fat and betaine supplements did not result in a clear benefit in terms of milk production or body temperature. Further work is warranted to understand the interactions between diet and hot weather.

## 1. Introduction

High ambient temperature and humidity can result in both short- and long-term effects on the milk production, reproduction and health of dairy cattle [1,2,3]. This is important for the dairy industry given the intensity, frequency and duration of heat waves are forecast to increase [4].

Feed intake of dairy cows is driven in part by their milk production [5]. High-producing cows are more susceptible to hot weather than low-producing cows due to greater internal heat production resulting from the digestion and metabolism of large amounts of feed eaten [3]. Hot weather causes a reduction in milk yield, with up to 50% of this reduction attributed to reduced dry matter intake (DMI) [6,7]. 

Increasing the fat concentration in the diet of dairy cows has been proposed as a way of reducing the heat load of cows by enabling them to have lower body temperatures during periods likely associated with heat stress [8]. Compared to other feeds, fat has a lower heat increment—the heat produced as a result of its digestion and metabolism [3,8]. The gross-energy density of fat (~39 MJ/kg DM) is greater than that of either protein (~24 MJ/kg DM) or carbohydrate (~18 MJ/kg DM) [9]. The gross-energy density of most grazed and conserved forages in Australia during summer is ~18 MJ/kg DM, with fat concentration in forages generally being less than 40 g/kg DM [10]. In contrast, canola oil contains approximately 990 g fat/kg [11]. Thus, supplementing a forage-based diet with a fat supplement can increase the energy density of the diet without reducing ruminal fermentation or voluntary DMI, providing the dietary fat concentration does not exceed 70 g/kg DM [12]. Furthermore, dairy cows offered high-fat diets during hot weather produced more energy-corrected milk (ECM) than cows offered low-fat diets [13,14]. This suggests that diets containing a high concentration of fat may reduce the negative impact that heat events have on milk production, while also limiting increases in body temperature due to lower heat of fermentation, digestion and metabolism of feed. 

Addition of betaine to the diet of animals can increase their resilience to heat exposure, with several modes of action on metabolism having been proposed [15]. Betaine (trimethylglycine) is a modified amino acid with three methyl groups that can act as methyl donors in various metabolic pathways. Observed effects when added to the diets of animals include reducing the incidence or severity of dehydration, reducing osmotic damage to tissues, reducing intracellular accumulation of Na+, reducing energy depletion within cells, and reducing protein denaturation. A reduction in each of these mechanisms reduces cellular damage and, perhaps most importantly, reduces the chance of endotoxins escaping from the gut into the body [15]. Betaine has been fed to sheep [16], dairy cattle [17,18,19], rabbits [20], and beef cattle [21], experiencing heat events with a variety of responses. There is little evidence that betaine directly offsets the physiological effects of hot conditions, but an animal with reduced organ and cellular damage is expected to recover faster once the heat load is removed. No reports were found detailing the effects of feeding betaine in the recovery period after heat exposure. 

Feeding fat and betaine together could be one way to maintain the milk production of cows during hot weather and minimize the physiological effects of heat stress. We were unable to find any reports of feeding a fat supplement in combination with betaine to ruminants. However, there is evidence from studies using mice that offering betaine in a high-fat diet is beneficial [22,23]. Offering cows a fat supplement during periods of heat exposure is expected to improve milk production [13,14] and reduce body temperature [8]. Offering betaine is also expected to improve milk production [17] and reduce body temperature [16]. Therefore, it is plausible that the effects of feeding fat and betaine together are additive. 

The objectives of this research were to determine the effects of adding canola oil (as an example fat) or betaine or canola oil plus betaine to the diet, on the DMI, milk production and body temperature of lactating dairy cows exposed to heat.

We hypothesized that compared to a non-supplemented diet, (1) supplementing the diet of dairy cows with fat before and during a heat challenge would result in smaller declines in DMI and ECM yield and a smaller increase in body temperature during the heat challenge, and (2) supplementing the diet of dairy cows with betaine before and during a heat challenge would result in smaller declines in DMI and ECM yield and a smaller increase in body temperature during the heat challenge. We also hypothesized that (3) during a heat challenge, cows given fat and betaine in combination would exhibit greater DMI and ECM yield and lower body temperature than cows given either alone. 

## 2. Materials and Methods

The experiment was conducted at the Agriculture Victoria Research, Ellinbank Research Centre, Victoria, Australia (38°14′ S, 145°56′ E).

### 2.1. Cows and Diets

Thirty-two multiparous, lactating Holstein-Friesian cows producing 18.6 ± 2.37 kg milk/d (mean ± standard deviation) with 566 ± 47.1 kg body weight (BW), 216 ± 18.5 days in milk (DIM), 2.7 ± 0.79 parity and 101 ± 2.9 heat tolerance breeding value (DataGene, Bundoora, Victoria, Australia; 100 = national breed mean) were assigned one of 4 diets on a daily basis (1) BASE—basal diet only, (2) CAN—basal diet plus 0.7 kg canola (*Brassica napus* L.) oil, (3) BET—basal diet plus 16 g betaine (trimethylglycine as a powder; Feedworks, Romsey, Victoria, Australia), or (4) CB—basal diet plus 0.7 kg canola oil and 16 g betaine (trimethylglycine). The daily basal diet was a total mixed ration (TMR) comprised of 7 kg DM lucerne hay (*Medicago sativa* L.), 6 kg DM pasture silage (predominantly perennial ryegrass, *Lolium perenne* L.), 5.0 kg DM grain mix (500 g/kg wheat grain (*Triticum aestivum* L.), 500 g/kg barley grain (*Hordeum vulgare* L.)), 1.5 kg DM solvent-extracted canola meal, 0.2 kg DM of minerals and vitamins (Ca 134 g/kg, Mg 110 g/kg, P 60 g/kg, Zn 6.4 g/kg, Mn 2.4 g/kg, Cu 1.2 g/kg, I 80 mg/kg, Co 100 mg/kg, Se 24 mg/kg, Vitamin A 165 IU/g, Vitamin D3 24 IU/g, Vitamin E 800 mg/kg), 0.1 kg DM salt, and 42 mL of Bloat Drench (271 g/L alcohols, C12-15 ethoxylated; VicChem, Coolaroo, Victoria, Australia). The compositions of the main dietary ingredients are shown in Table 1. Betaine doses were wrapped in ~50 g DM of silage, then offered by hand to individual cows on betaine treatments prior to the bulk of the ration being offered. Canola oil was incorporated into the CAN and CB rations by pouring it over the feed and mixing the ration by hand. 

### 2.2. Experiment Design

All cows were familiarized with the experimental environments prior to the experiment by feeding them in each of those environments for seven days. The cows were managed in four cohorts of eight cows. Each treatment was assigned at random to two cows within each cohort, while simultaneously balancing treatment groups for BW, milk yield, DIM and heat tolerance breeding value using covariate design software in Genstat 19 (VSN International Ltd., Hemel Hempstead, UK). Six of the eight cows in each cohort were assigned to six controlled-climate chambers for heat challenge according to a row-column design with cohorts as rows and chambers as columns. Each diet treatment appeared once in each chamber and once or twice in each cohort. The two cows not exposed to the heat challenge in each cohort were selected as a different pair of treatments in each cohort and were available as spares for use in the event of loss for any extraneous reason, such as mastitis or lameness. Cohorts were staggered in time to ensure that all animals entered the heat challenge after the same number of days on the diet.

During the covariate period (days 1 to 7) cows were managed as a single group under ambient conditions in a paddock with measurements made of milk yield and composition, and BW. During this time, cows were offered 5 kg DM/day of crushed wheat grain during milking and approximately 15 kg DM/day of lucerne hay per day in a paddock with negligible pasture available.

The adaptation period was commenced on day 8. Cows were moved to the experiment facilities where they were fed in individual feed stalls for two periods of 3.5 h each within a well-ventilated animal house [24] and rested on a loafing pad covered with wood chips. Cows were transitioned to their treatment diets over three days (days 8 to 10), then adapted to those diets for a further 11 days (days 11 to 21). 

Pre-challenge measurements were recorded for three days (days 22 to 24) while cows were housed in ambient conditions. 

The heat challenge was conducted for up to four days (days 25 to 28). Six cows in each block were individually exposed to the heat challenge in controlled-climate chambers [25]. During the heat challenge, the targeted conditions were 30 °C and 50% RH (temperature-humidity index, THI = 80) from 0601 to 1200 h; 33 °C and 50% RH (THI = 84) from 1201 to 1800 h; and 25 °C and 60% RH (THI = 74) between 1801 to 0600 h. If an individual cow’s rectal temperature was greater than 40.9 °C (the limit approved by the animal ethics committee), the cow was cooled by opening the chamber doors and adjusting the conditions in the chamber to thermoneutral (17 °C, 60% RH). Cows were also cooled if, in the opinion of the investigators, continuing in the heat challenge would be detrimental to the health of the cow. Individual cows thus cooled stayed within their chamber at thermoneutral conditions for the remainder of their scheduled time in the chamber. Only data from cows completing two or more days of the heat challenge were included in the analysis.

Cows that were cooled had their recovery monitored from the day chamber temperature was reduced. The recovery of cows completing the four-day heat challenge was monitored under ambient conditions for 7 d (days 29 to 35). 

### 2.3. Feeding and Feed Analysis

Feed was offered in two equal portions immediately following the morning and afternoon milkings. Dry matter concentration was determined on samples of grain mix, canola meal and minerals collected on two consecutive days of each week; samples of lucerne hay were collected every morning and silage at every feeding. Refusals of the TMR were collected, weighed and sampled. Dry matter concentration was determined by drying in a forced draft oven at 105 °C for 24 h. 

The nutritional composition was determined on samples of the grain mix, canola meal, lucerne hay, and silage offered that were collected daily, a 100 g sub-sample was taken, bulked by week for each feed type, and stored at −18 °C. Bulked samples were subsequently freeze-dried and ground to pass through a 0.5-mm screen then analyzed for crude protein (CP), soluble protein, acid detergent fiber (ADF), neutral detergent fiber (NDF), lignin, non-fiber carbohydrate, starch, ash, total digestible nutrients (TDN), crude fat (ether extract, EE), sodium, potassium, calcium, magnesium, phosphorous, sulfur and chloride by chemical analytical methods according to the procedures of Dairy One [26]. 

Water was offered to all animals at least once during each 3.5 h feeding period and was available ad libitum when cows were on the loafing pad or in the controlled-climate chambers.

### 2.4. Milk Production and Composition 

Cows were milked twice daily, at ~0600 h and ~1500 h. Throughout the experiment, milk yield was measured for each cow at each milking. When cows were not housed in the chambers, milk yields were recorded automatically using a DeLaval milk metering system (MM25; DeLaval International, Tumba, Sweden). Milk samples for composition analysis were collected on days 1 to 7 (covariate), 1 of days 22 to 24 (pre-challenge, day closest to thermoneutral), and days 29 to 35 (recovery). During the heat challenge in the chambers, milk yield measurements were made by collecting and weighing the milk from individual cows and milk samples were collected at every milking. Fat and protein in milk samples were measured by means of a near-infrared milk analyzer (model 2000, Bentley Instruments, Chaska, MN, USA). Energy-corrected milk, standardized to 4.0% fat and 3.3% protein, was calculated using Equation (1) [27]:

ECM (kg/d) = (milk yield (kg) × (376 × fat% + 209 × protein% + 948))/3138,(1)

### 2.5. Physiology

Body temperature was measured intravaginally (vaginal temperature) and recorded every 15 min during the covariate, pre-challenge, heat challenge and recovery periods using temperature loggers (iButton DS1922L; Maxim Integrated, San Jose, CA, USA) as described by Garner et al. [25]. Rectal temperature was only measured during the heat challenge at 0600 h and 1500 h using an EcoScan Temp 5 with 100 K thermistor temperature probe (Eutech Instruments, Singapore, Singapore).

### 2.6. Calculations and Statistical Analyses

Temperature-humidity index (THI) was calculated using Equation (2) [28].

THI = Tdb + (0.36 × Tdp) + 41.2,(2)

where:

Tdb is dry bulb temperature (°C);
Tdp is dew point temperature (°C),
Tdp = (237.3 × b)/(1.0 − b);
b = [log(RH/100.0) + (17.27 × Tdb)/(237.3 + Tdb)]/17.27.
RH = relative humidity (%)

Temperature and relative humidity data were collected every 10 min during the pre-challenge and recovery periods using Minnow 1.0 loggers (Senonics LLC, Arvada, CO, USA). During the heat challenge, temperature and relative humidity data were recorded every 1 min by the control system of the controlled-climate chambers. A THI threshold of 68 was used to determine if weather conditions were imposing heat stress on the animals as this is the point that has previously been identified as when weather conditions start affecting milk production [29].

Pre-challenge data for analysis were intended to represent thermoneutral conditions and were recorded under ambient conditions on a day just prior to the heat challenge. 

The proportion of cows completing all four days of the heat challenge was tested using a generalized linear model with change in deviance χ^2^ tests [30]. The model used binomial distribution for the number completing out of the number eligible, with log-link function and factorial effects of fat by betaine. 

There were two types of statistical analyses conducted on experimental data: (1) an analysis of the main effect of the heat challenge or time, (2) the factorial effects of canola oil and betaine diet within periods or on changes between periods, 

(1) Effect of heat challenge: Trends over time in DMI, milk production and body temperature averaged across diet treatments were assessed for all cows that completed the heat challenge. This was achieved by calculating summary statistics for each cohort since each cohort provided an independent replication with respect to time, and using them as data in a *t*-test on degrees of freedom. Summary statistics were calculated as mean changes between pre-challenge and heat challenge, heat challenge and recovery, or pre-challenge and recovery, and as linear regression slopes from the last day of pre-challenge to day 4 of heat challenge, and from the last day of heat challenge to day 7 of recovery. 

(2) Factorial effects of diet: Only data from cows that had completed two or more days of the heat challenge were included in the analysis of treatment effects. Data from 22 cows (BASE, *n* = 6; CAN, *n* = 4; BET, *n* = 6; CB, *n* = 6) from day 2 of the heat challenge were used to represent the effects of the heat challenge. This was a compromise between the heat challenge having an effect on the cows and retaining sufficient animals in the analysis to enable conclusions to be drawn. Variables were constructed to address hypotheses, with one datum per animal. To achieve this for each animal, raw data within the pre-challenge period were averaged, and day 2 of the heat challenge was taken to represent the heat challenge. In addition, change variables were calculated for each individual cow by taking the difference between the mean value of a specific variable measured during the pre-challenge period and the value of the same variable measured during day 2 of the heat period. Similar change variables were calculated between the first and second days of recovery (initial recovery), and between the pre-challenge period and the final day (day 7) of the recovery period (extent of recovery). Each of these constructed variables was subjected to statistical analysis using the following linear model:

*y* = μ + βy_cov_ + F + B + FB + C + Κ + ε, (3)

where *y* was the outcome variable of interest, y_cov_ was the same variable if available from the covariate period, F was a factor for the effect of added fat, B was a factor for the effect of added betaine and FB their interaction, C was an effect of chamber, K as an effect of cohort and ε a random error for individual animal. The model was applied using ReML software in GenStat 20 (VSN International Ltd., Hemel Hempstead, UK) with fixed effects for the covariate, cohort, chamber, and factorial fat by betaine diet treatments, and with animal as a random effect. Residuals were examined graphically to check distributional assumptions of normality and constant variance.

## 3. Results

### 3.1. Effect of Heat Challenge

**Figure 1:**
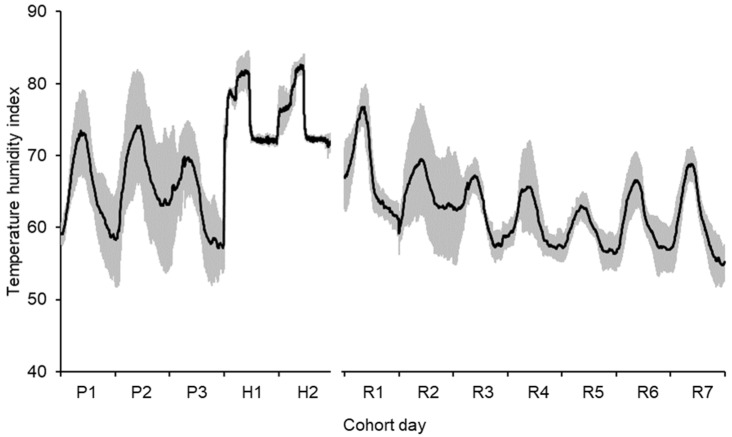
Mean environmental conditions experienced by the cows during the pre-challenge (P), heat challenge (H) and recovery periods (R) (grey band shows ± one standard deviation about the mean). The heat challenge was generated in controlled-climate chambers. Cows were in ambient conditions before and after the heat challenge.

Mean daily THI during the pre-challenge period was 65 ± 7.3 (mean ± standard deviation, Figure 1). Measurements were made and samples were collected only on days when maximum THI was less than 68. All cohorts experienced at least one day of ambient conditions with maximum THI greater than 68 in the pre-challenge period. 

During the heat challenge, mean daily THI in the controlled-climate chambers was 75 ± 4.1. Mean daily THI during the recovery period was 62 ± 5.6. All cohorts experienced at least two days of ambient conditions with a maximum THI greater than 68 during the recovery period.

**Figure 2:**
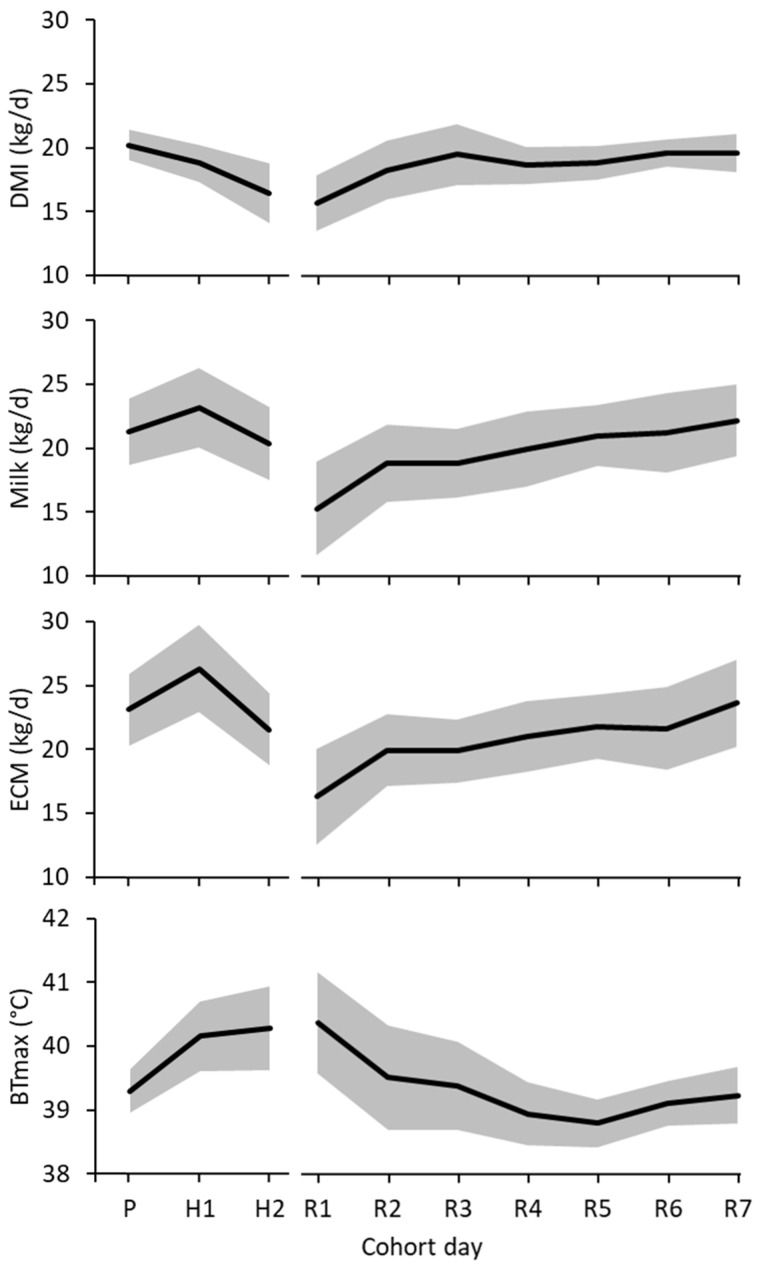
Mean daily dry matter intake (DMI), milk yield (Milk), energy corrected milk yield (ECM) and maximum vaginal temperature (BTmax) of all cows during the heat challenge (H) in controlled-climate chambers (grey band shows ± one standard deviation about the mean). Cows were in ambient conditions during the pre-challenge (P) and recovery (R) periods.

Across all treatments, the conditions during the heat challenge induced heat stress in all cows. This was evidenced by a reduction in daily DMI (3.9 ± 1.22 kg DM, *p* = 0.008) from the pre-challenge period to day 2 of the heat challenge (Figure 2) and an increase in maximum vaginal temperature (1.0 ± 0.24 °C, *p* < 0.004). For those cows that completed the 4-day heat challenge, DMI declined to a minimum on day 4 of the heat challenge then increased during the recovery period to 99% of pre-challenge at recovery day 7. Both milk yield and ECM were lowest on day 1 of the recovery, increased to around 90% of pre-challenge on recovery day 2 then increased to pre-challenge by recovery day 7.

### 3.2. Challenge Completion

Not all cows completed all four days of the heat challenge (Table 2). Two cows on the CAN diet were excluded from the analysis, one due to injury sustained on day 1 of the heat challenge, and one due to equipment failure that resulted in no heating on day 1 of the heat challenge. Of the remaining 22 cows, two cows (1 BASE, 1 CAN) were cooled on day 3, and seven cows (2 BASE, 1 CAN, 1 BET, 3 CB) were cooled on day 4 of the heat challenge because their body temperature exceeded the predetermined limit of 40.9 °C. One cow (CB) was cooled on day 4 of the heat challenge at the discretion of the investigators as she exhibited an extended period of the elevated panting score. There was no difference due to treatment on the number of cows completing the heat challenge (*p* = 0.234). However, it must be noted that the two cows on the CAN treatment lost on day 1 of the heat challenge were not due to treatment (1 physical injury, 1 equipment failure) and the remaining low number of animals meant the resultant power of this analysis was low. 

### 3.3. Main Effects-Dry Matter Intake

Dry matter intake during the pre-challenge period (Table 3) was not affected by the feeding of canola oil (*p* = 0.081) nor betaine (*p* = 0.181). Intake of metabolizable energy was greater in cows fed canola oil than in cows not fed canola oil (*p* < 0.001), which was in accordance with the experiment design. 

On day 2 of the heat challenge, DMI was 15% lower in cows given canola oil than other cows but this only tended towards being different (*p* = 0.071, Table 3). Feeding betaine had no effect on DMI (*p* = 0.489). There was no difference in intake of ME between treatments. 

From the pre-challenge period to day 2 of the heat challenge, the decline in DMI was greater for cows offered canola oil than those not offered canola oil (*p* = 0.035) and betaine had no effect (*p* = 0.68). The reduction in ME intake was greater for cows given canola oil compared with those not (*p* = 0.029). Offering betaine had no effect on the decline in ME intake (*p* = 0.875). 

During the initial recovery, DMI increased for all cows (*p* = 0.005). However, neither canola oil (*p* = 0.11) nor betaine (*p* = 0.765) had any effect. By day 7 of the recovery period, there was a tendency (*p* = 0.07) for a decrease in DMI of 3.8 kg/d for all cows relative to the pre-challenge period. This change was unaffected by the canola oil (*p* = 0.45) or betaine (*p* = 0.49) diets.

### 3.4. Main Effects-Milk Yield

During the pre-challenge period, milk yields (Table 4) were greater from cows fed canola oil than from cows not fed canola oil (*p* < 0.001) and similarly for ECM (*p* = 0.032). Cows fed canola oil also had greater yields of protein (*p* = 0.007) but not fat (*p* = 0.138) compared to the yields from cows not fed canola oil. Cows fed canola oil produced milk with lower concentrations of milk fat (*p* = 0.022) compared to those not fed canola oil. Cows fed betaine tended to have lower milk yield than those not fed betaine (*p* = 0.073).

On day 2 of the heat challenge, milk yield was greater from cows given canola oil than those not (*p* = 0.026), but ECM yield was only numerically greater (*p* = 0.105). 

From the pre-challenge period to day 2 of the heat challenge, the decline in milk yield was not affected by feeding canola oil (*p* = 0.429) or betaine (*p* = 0.540). The decline in ECM yield was unaffected by either canola oil (*p* = 0.778) or betaine (*p* = 0.429). 

During the initial recovery, milk yield increased for all cows. The increase in milk yield was not affected by the feeding of either canola oil (*p* = 0.823) or betaine (*p* = 0.610). Yield of ECM also increased for all cows but feeding neither canola oil nor betaine had an effect. 

Recovery of ECM yield to seven days post-heat challenge was unaffected by treatment and there was no interaction between canola oil and betaine. 

### 3.5. Main Effects-Body Temperature

During the pre-challenge period, the mean vaginal temperature was not affected by the addition of canola oil to the diet, or the supplementation with betaine (Table 5). However, the maximum vaginal temperature of cows fed betaine was lower than that of cows not fed betaine (*p* = 0.014). 

On day 2 of the heat challenge, cows fed canola oil had greater mean (*p* = 0.016) and maximum vaginal temperatures (*p* = 0.039) than other cows. Feeding betaine had no effect on mean (*p* = 0.31) or maximum (*p* = 0.37) vaginal temperatures. 

From the pre-challenge period to day 2 of the heat challenge, cows given canola oil tended to have a greater increase in mean vaginal temperature (*p* = 0.074) than other cows but there was no effect on maximum vaginal temperature (*p* = 0.127). Supplementing the cows’ diet with betaine had no effect on the change in mean (*p* = 0.679) or maximum (*p* = 0.825) body temperature.

During the recovery period, the mean vaginal temperature decreased in all cows from day R1 to R2 (Table 5) with the change tending to be greater in cows fed canola oil than other cows (*p* = 0.07). The change in maximum vaginal temperature from day R1 to R2 was not affected by either canola oil (*p* = 0.38) or betaine (*p* = 0.60). Neither canola oil nor betaine had any effect on the difference in vaginal temperature between day R7 and pre-challenge (*p* > 0.38), and this small difference in vaginal temperature was not significantly different to zero (*p* > 0.16).

### 3.6. Treatment Effects—Dry Matter Intake

On day 2 of the heat challenge, the DMI of cows given the CAN diet was less than that of cows given the BASE diet (*p* < 0.05), but there was no difference in DMI between cows given the CB and BASE diets. Similarly, the decline in DMI from the pre-challenge period to day 2 of the heat challenge in cows offered the CAN diet was greater than that in cows offered the BASE diet (*p* < 0.05), but there was no difference in decline of DMI between cows given the CB and BASE diets.

### 3.7. Treatment Effects—Milk Yield

From the pre-challenge period to day 2 of the heat challenge, the change in milk yield of cows on the BET diet was +0.3 kg/day, but this was not statistically different (*p* > 0.05) from the change in milk yield of cows on the other diets (−1.1 kg/day). Dietary treatment had no effect on the declines in yield of fat (*p* > 0.330), or protein (*p* > 0.490). Moreover, dietary treatment did not influence (*p* > 0.300) the change in composition of milk fat or milk protein.

During the initial recovery, there was a tendency for an antagonistic interaction (*p* = 0.054) between canola oil and betaine for milk yield such that the increase in milk yield for cows given the CB diet was 2.5 kg/day compared with 4.2 ± 0.82 kg/day for each of the CAN and BET treatments and 1.3 kg/day for cows on the BASE diet.

### 3.8. Treatment Effects—Body Temperature

On day 2 of the heat challenge, mean vaginal temperature of cows offered the BET treatment was lower than that of cows offered the CAN and CB treatments. However, none of these treatments resulted in a mean vaginal temperature different to that of the cows offered the BASE diet.

## 4. Discussion

The temperature and humidity settings applied in the controlled-climate chambers successfully induced a state of heat stress in all cows. This was observed as an 18% decrease in DMI and a 1 °C increase in maximum vaginal temperature of the cows. However, the heat challenge was not completed by all cows. While challenge completion may have been influenced by treatment, we were unable to detect any treatment effect. Ten of our 24 cows were removed from the heat challenge on either day 3 or 4. For this reason, we chose to analyze the effects of treatment at the completion of day 2 of the heat challenge using data from the 22 cows that completed the heat challenge to this point. 

### 4.1. Canola Oil Main Effect

Cows offered diets supplemented with canola oil had a greater decline in DMI, a similar decline in ECM, and a greater increase in body temperature than cows not offered canola oil. Thus, we reject our first hypothesis. 

During the pre-challenge period, our cows offered diets containing canola oil had a 7% greater intake of metabolizable energy compared to cows offered diets not containing canola oil. However, during the heat challenge, the large decrease in DMI of the cows given canola oil meant that their resulting intake of metabolizable energy was 7% less than that of the other cows. Thus, the greater concentration of energy in the canola oil diets did not offset the reduction in intake of metabolizable energy due to the reduction in DMI. Why the DMI of cows offered canola oil declined so dramatically is not known. Fat supplementation has been shown to reduce voluntary DMI due to the depression of ruminal fiber digestion [31]. However, in our experiment the canola oil supplement was added to the basal diet instead of being substituted for the existing components of the diet, and the total concentration of fat in the canola oil supplemented diets was below the threshold of 70 g/kg DM where DMI can be depressed [12]. Furthermore, the fatty acids in canola mainly comprise oleic and linoleic acids [32], and almost none of the medium chain fatty acids (lauric and myristic) that have generally been associated with inhibition of dry matter intake [33,34]. 

The effects of dietary fat supplementation on ECM during hot weather are generally positive. In agreement with our results, dairy cows fed fat during hot weather generally have greater production of milk and ECM relative to those cows not receiving the supplement [13,14,35]. Experiments using controlled-environment chambers to create a heat challenge also found greater milk yields in cows fed a diet supplemented with fat [36,37]. However, some studies report nil effect of dietary fat on milk production during hot weather [38]. This could be due to a number of reasons, such as the type of fat supplement [39], the severity and duration of heat exposure, and in the case of Moallem et al. [38], the use of cooling amenities five times per day. 

Our cows offered canola oil produced 3.5 kg more milk (2.8 kg ECM) per cow per day during the pre-challenge period than those cows not offered canola oil. The increase in milk is explained by the additional energy intake derived from the canola oil supplement, and the difference between milk yield and ECM yield is explained by the observed decrease in milk fat concentration during the pre-challenge period. This can occur in cows offered a diet with a fat supplement due to the formation of fatty-acid isomers in the rumen that cause milk fat depression [31].

Some previous reports agree with our findings on body temperature in which dairy cows fed a dietary fat supplement during hot weather had greater body temperature than the cows that were not fed the fat supplement [38]. This may have contributed to the reduction in feed intake in the CAN treatment. Conversely, Drackley et al. [13] and Huber et al. [35] reported no effect of dietary fat supplementation on rectal temperature of dairy cows during hot summer conditions, and studies with cows subjected to heat-stress conditions in environmental chambers also showed no difference [36,37]. These findings, and ours, are contrary to the expectation that feeding fat to cows would result in lower body temperature due to the lower heat increment of fat compared to other feeds [3,8]. We could not find evidence to suggest that these differences might be due to the fatty acid profile, or specific fatty acids, of the fats reported. However, Wang et al. [39] found that offering a fat supplement with a high proportion of saturated fatty acids resulted in lower body temperatures compared with no fat supplementation, but only when measurements were made during the hottest part of the day. It is possible that body temperature is more closely related to energy intake than feed type. 

Results from our pre-challenge period agree with a previous report that dietary addition of fat had no effect on the rectal temperature of lactating cows during thermoneutral conditions [37]. We note that previous studies that have examined the effects of dietary supplementation with fat [13,17,37] have generally not continuously measured body temperature.

### 4.2. Betaine Main Effect

Cows offered a diet supplemented with betaine did not have smaller declines in DMI or ECM than cows not offered betaine, and the increase in body temperature was also not different during a heat challenge. Thus, we reject our second hypothesis. 

Our DMI results are consistent with previous research in which betaine was offered to dairy cows at low inclusion rates [40]. In addition, betaine fed in the range of 50 to 150 mg/kg BW^0.75^ to beef heifers in ambient conditions had no effect on DMI [41]. Similarly, the DMI of sheep fed betaine during a heat challenge was not different from other animals [16]. In contrast, one study reported the DMI of dairy cows fed betaine during a heat challenge was lower than those not fed a betaine supplement [17]. However, the betaine dose of 563 mg/kg BW^0.75^ used [17] was much greater than the 132 mg/kg BW^0.75^ used in our experiment and greater than the optimum dose of 126 mg/kg BW^0.75^ reported for sheep [16]. 

While there was no increase in milk yield during our short-term heat challenge, greater yields of milk have been reported previously in cows offered similar doses of betaine to that used in our experiment [42,43]. It has been suggested that the greatest effect of betaine may be observed when the overnight temperature remains high [43]. Our betaine dose was in the range suggested by Dunshea et al. [43] to be optimal for beneficial effects but it is possible the overnight temperature during the heat challenge in our experiment (25 °C) was too low, or the heat challenge too short, for the benefits of betaine supplementation to be realized. 

The body temperatures of our cows during the heat challenge are similar to those in previous research where the diet of lactating dairy cows was supplemented with betaine at 57 and 114 mg/kg BW^0.75^ for 14 days before cows were exposed to a heat challenge [17], and 17 to 35 mg/kg BW^0.75^ for three months of summer [18]. However, supplementing the diet of sheep with betaine at 126 mg/kg BW^0.75^ during heat exposure for three weeks has been found to reduce rectal temperature [16]. Furthermore, a study in buffalo heifers under heat-stress conditions showed that dietary inclusion of betaine at approximately 420 mg/kg BW^0.75^ was associated with decreased rectal temperature, but the effect was not statistically significant until the fifth week of the experiment [44]. With the exception of the report by Zhang et al. [18], who pooled their results across time, there appears to be a correlation between the duration of feeding of betaine and the extent of the treatment effect. This concept is further supported by a report of the effect of betaine increasing with time when fed to lactating dairy cows over summer [43]. In our experiment, betaine was fed to cows for 14 days prior to the heat challenge. Therefore, we speculate that in our experiment, the short duration of inclusion of betaine in the diet may be one reason why we did not observe an effect on body temperature during the heat challenge.

During the initial recovery (day R1 to R2), cows given betaine did not show a greater increase in DMI compared to other cows but their increase in ECM was numerically double that observed in the other cows. However, not all cows completed the heat challenge. This means that the heat challenge was of different lengths for different cows, thereby confounding our assessment of the recovery. Previous reports indicated that betaine should have reduced the effects of the heat challenge on our cows [15] leaving them better positioned to recover once conditions returned to thermoneutral. Reports of animals fed betaine performing better during a heat challenge [42,44] also support the idea that the effects of the heat challenge are lessened for those animals, and as a corollary they should be better positioned to recover. No reports of dairy cow performance immediately following a heat challenge were found in the scientific literature. 

### 4.3. Supplement Interaction–Treatment Effect

Cows given the fat and betaine in combination did not exhibit greater DMI and ECM yield, and lower body temperature during a heat challenge compared to cows given either alone. Thus, we reject our third hypothesis. However, the variability in results during the heat challenge did reduce our ability to detect differences. On day 2 of the heat challenge the DMI of cows given the CAN diet was less than that of cows given the BASE diet, but there was no difference in DMI between cows given the CB and BASE diets. This suggests that betaine could offset some of the negative impact that canola oil had on DMI in our experiment. A similar effect on body temperature was not observed. Cows given the BET diet did have lower mean and maximum body temperature than the cows given the CAN diet, but those given the CB diet had temperatures close to those given the CAN diet. 

We speculated that the concentration of fat in the diet could have influenced the reported animal responses to dietary betaine. However, a re-examination of the scientific literature used to determine the betaine dose for our experiment found that only four of the 10 reports specified the concentration of fat in the basal diets, and therefore we were unable to draw a conclusion. In our experiment, the small number of cows in each individual treatment meant that an in-depth investigation of the interactions was not possible, so further work is warranted to untangle the interactions between dietary fat and betaine supplements.

## 5. Conclusions

Cows subjected to a heat challenge had decreased DMI and milk production with a concomitant increase in vaginal temperature. Neither canola oil nor dietary betaine supplements had the expected effects on cow milk production or body temperature during a heat challenge. Supplementing the diet of dairy cows with fat resulted in a positive milk production response, and the magnitude of the response was similar before, during and after a heat challenge. However, dietary fat supplementation also resulted in greater body temperature across all periods of our experiment. The declines in DMI and ECM from pre-challenge to the heat-challenge were greater in cows fed canola oil, in contrast to expectations that the declines would be reduced when canola oil was fed. Contrary to our expectations, the combination of fat and betaine supplements did not result in any clear benefit in terms of milk production or body temperature. Due to the short duration of the heat challenge and a low number of cows in our experiment, further research is required to fully understand the interactions between dietary fat type and betaine supplements when offered to cows during periods of hot weather.

## Figures and Tables

**Table 1:** Composition of main dietary ingredients (g/kg DM unless otherwise stated).

Parameter	Grain Mix ^1^	Lucerne Hay	Pasture Silage
Crude protein	194	168	176
Soluble protein (% CP)	26.6	39.7	65.6
Acid detergent fiber	73	364	336
Neutral detergent fiber	153	457	508
Lignin	20	78	36
Non-fiber carbohydrate	586	278	154
Starch	486	16	5
Ash	40	78	110
Total digestible nutrients	818	583	642
Calcium	2.2	8.7	5.8
Magnesium	2.3	2.1	2.0
Sodium	0.2	0.8	3.7
Potassium	6.2	24.0	35.5
Chloride	1.1	6.7	11.9
DCAD (meq./100 g DM)	−4.04	29.4	54.2
Copper (mg/kg DM)	6.46	7.43	6.39
Sulfur	2.8	2.6	3.1
Crude fat	27.5	18.6	51.4
ME ^2^ (MJ/kg DM)	13.7	9.4	10.5

^1^ Grain mix consisted of 500 g/kg wheat grain, 500 g/kg barley grain. ^2^ ME = metabolizable energy.

**Table 2:** Number of cows completing each day of the heat challenge.

Diet ^1^	Cows Entering Challenge	Day of Heat			
		1	2	3	4
BASE	6	6	6	5	3
CAN	6	4	4	3	2
BET	6	6	6	6	5
CB	6	6	6	6	2

^1^ BASE = control diet, CAN = control plus canola oil, BET = control plus betaine, CB = control plus canola oil and betaine.

**Table 3:** Intake of dry matter (DMI) and metabolizable energy (MEI) during selected periods of the experiment.

	Main Effects									Treatment Effects				
	Can ^1^		Bet		SEDm ^2^		*p*-Value							
	−	+	−	+	Can	Bet	Can	Bet	Can × Bet	BASE ^3^	CAN	BET	CB	SEDt ^4^
*n*	12	10	10	12						6	4	6	6	
Pre-challenge														
DMI	20.1	20.4	20.1	20.4	0.20	0.21	0.081	0.181	0.181	20.1 ^a^	20.2 ^ab^	20.1 ^a^	20.7 ^b^	0.29
MEI	224	239	230	233	2.2	2.2	0.001	0.306	0.293	224 ^a^	237 ^b^	224 ^a^	242 ^b^	3.1
Heat challenge day 2														
DMI	17.4	14.9	15.7	16.6	1.07	1.08	0.071	0.489	0.153	17.8 ^b^	13.6 ^a^	17.0 ^ab^	16.2 ^ab^	1.53
MEI	194	181	185	190	11.8	11.9	0.307	0.716	0.284	199	171	190	190	16.9
Pre challenge to heat														
ΔDMI ^5^	−2.7	−5.4	−4.4	−3.8	1.03	1.04	0.035	0.680	0.235	−2.3 ^b^	−6.4 ^a^	−3.1 ^ab^	−4.5 ^ab^	1.47
ΔMEI	−29	−58	−45	−43	10.9	11.0	0.029	0.875	0.367	−26 ^b^	−64 ^a^	−34 ^ab^	−51 ^ab^	15.5
Initial recovery														
ΔDMI ^6^	3.3	1.9	2.8	2.3	1.00	1.01	0.113	0.765	0.133	2.7 ^ab^	3.0 ^ab^	3.9 ^b^	0.8 ^a^	1.44
ΔMEI	37	27	36	27	10.6	10.7	0.288	0.465	0.102	31.3	40.8	41.7	12.2	15.2

^1^ Main effects: Can = canola oil, Bet = betaine; ^2^ SEDm = standard error of the difference between main effects; ^3^ Treatment diet effects: BASE = basal diet, CAN = BASE plus canola oil, BET = BASE plus betaine, CB = BASE plus canola oil and betaine; ^4^ SEDt = standard error of the difference between treatments; ^5^ Δ variable = (heat challenge day 2 variable—Pre-challenge variable); ^6^ Δ variable = (Recovery day 2 variable—Recovery day 1 variable); ^a,b^ Treatment means with different superscripts are different (*p* < 0.05).

**Table 4:** Milk yield (kg/d) and composition (g/kg) during selected periods of the experiment.

	Main Effect									Treatment Effects				
	Can ^1^		Bet		SEDm ^2^		*p*-Value							
	−	+	−	+	Can	Bet	Can	Bet	Can × Bet	BASE ^3^	CAN	BET	CB	SEDt ^4^
*n*	12	10	10	12						6	4	6	6	
Pre-challenge														
Milk yield	19.8	23.3	22.2	20.9	0.60	0.62	0.001	0.073	0.658	20.3 ^a^	24.0 ^b^	19.3 ^a^	22.5 ^b^	0.87
ECM^4^ yield	21.7	24.5	23.9	22.3	1.04	1.11	0.032	0.255	0.261	21.8 ^a^	25.9 ^b^	21.6 ^a^	23.1^ab^	1.52
Fat yield	0.93	1.02	1.02	0.93	0.05	0.05	0.138	0.172	0.106	0.93 ^a^	1.11 ^b^	0.93 ^a^	0.93 ^a^	0.07
Protein yield	0.70	0.80	0.77	0.73	0.03	0.03	0.007	0.305	0.313	0.70 ^a^	0.84 ^b^	0.70 ^a^	0.77 ^ab^	0.04
Fat concentration	47.7	44.1	45.9	46.0	1.4	1.4	0.022	0.891	0.346	47.0 ^ab^	44.7 ^ab^	48.5 ^b^	43.5 ^a^	1.94
Protein concentration	35.8	34.9	35.0	35.7	0.68	0.69	0.227	0.350	0.658	35.3	34.7	36.3	35.1	0.96
Heat challenge day 2														
Milk yield	19.4	22.2	21.2	20.4	1.03	1.06	0.026	0.525	0.268	19.1 ^a^	23.2 ^b^	19.6 ^a^	21.2 ^ab^	1.49
ECM yield	20.8	23.2	22.4	21.6	1.30	1.39	0.105	0.716	0.259	20.3	24.4	21.2	22.0	1.91
Fat yield	0.88	0.96	0.94	0.90	0.06	0.07	0.265	0.751	0.246	0.86	1.02	0.91	0.90	0.09
Protein yield	0.65	0.75	0.71	0.68	0.04	0.04	0.036	0.555	0.187	0.64 ^a^	0.79 ^b^	0.67 ^a^	0.70 ^ab^	0.05
Fat concentration	45.4	43.5	44.1	44.9	1.71	1.76	0.291	0.643	0.780	44.8	43.3	46.1	43.7	2.4
Protein concentration	33.9	33.6	33.8	33.7	0.52	0.52	0.467	0.996	0.754	33.9	33.6	34.0	33.5	0.73
Pre challenge to heat														
∆Milk yield ^5^	−0.4	−1.1	−1.0	−0.5	0.83	0.85	0.429	0.540	0.288	−1.1	−0.8	0.3	−1.3	1.20
∆ECM yield	−1.0	−1.3	−1.5	−0.7	0.93	0.99	0.778	0.429	0.739	−1.5	−1.5	−0.4	−1.1	1.37
∆Fat yield	−0.05	−0.06	−0.08	−0.03	0.05	0.05	0.974	0.335	0.901	−0.07	−0.09	−0.03	−0.02	0.07
∆Protein yield	−0.04	−0.06	−0.06	−0.05	0.02	0.02	0.491	0.617	0.382	−0.06	−0.05	−0.03	−0.06	0.03
∆Fat concentration	−2.3	−0.6	−1.8	−1.1	1.64	1.68	0.304	0.710	0.613	−2.2	−1.4	−2.4	0.2	2.3
∆Protein concentration	−1.9	−1.4	−1.3	−2.0	0.62	0.63	0.453	0.309	0.821	−1.5	−1.1	−2.3	−1.6	0.88
Initial recovery														
∆Milk yield ^6^	2.7	3.3	2.7	3.3	0.99	1.01	0.823	0.610	0.054	1.3	4.2	4.2	2.5	1.43
∆ECM yield	3.5	3.6	2.3	4.8	1.32	1.46	0.655	0.134	0.293	1.4	3.3	5.7	3.9	2.11
∆Fat yield	0.16	0.14	0.08	0.23	0.08	0.08	0.863	0.131	0.320	0.05	0.11	0.28	0.17	0.12
∆Protein yield	0.12	0.13	0.09	0.15	0.03	0.04	0.460	0.152	0.612	0.08	0.11	0.16	0.15	0.05
∆Fat concentration	1.2	−1.0	−3.5	3.7	4.0	4.6	0.820	0.165	0.680	−3.49	−3.53	5.90	1.48	6.68
∆Protein concentration	−0.58	0.07	−0.99	0.48	0.54	0.63	0.182	0.054	0.620	−1.14 ^a^	−0.83 ^ab^	−0.02 ^ab^	0.98 ^b^	0.91

^1^ Main effects: Can = canola oil, Bet = betaine; ^2^ SEDm = standard error of the difference between main effects; ^3^ Treatment diet effects: BASE = basal diet, CAN = BASE plus canola oil, BET = BASE plus betaine, CB = BASE plus canola oil and betaine; ^4^ SEDt = standard error of the difference between treatments; ^5^ Δ variable = (heat challenge day 2 variable—Pre-challenge variable); ^6^ Δ variable = (Recovery day 2 variable—Recovery day 1 variable); ^a,b^ Treatment means with different superscripts are different (*p* < 0.05).

**Table 5:** Vaginal temperature (°C) during selected periods of the experiment.

	Main Effects									Treatment Effects				
	Can ^1^		Bet		SEDm ^2^		*p*-Value							
	−	+	−	+	Can	Bet	Can	Bet	Can × Bet	BASE ^3^	CAN	BET	CB	SEDt ^4^
*n*	12	10	10	12						6	4	6	6	
Pre-challenge														
Mean	38.3	38.4	38.4	38.3	0.08	0.08	0.477	0.227	0.510	38.3	38.5	38.3	38.3	0.11
Maximum	39.4	39.4	39.6	39.2	0.10	0.11	0.530	0.014	1.000	39.5 ^b^	39.6 ^b^	39.2 ^a^	39.2 ^ab^	0.15
Heat challenge day 2														
Mean	39.0	39.6	39.4	39.2	0.19	0.20	0.016	0.314	0.614	39.2 ^ab^	39.6 ^b^	38.9 ^a^	39.5 ^b^	0.27
Maximum	40.0	40.5	40.4	40.1	0.25	0.28	0.039	0.371	0.789	40.1 ^ab^	40.6 ^b^	39.8 ^a^	40.5 ^ab^	0.38
Pre challenge to heat														
ΔMean ^5^	0.74	1.18	1.01	0.91	0.21	0.22	0.074	0.679	0.487	0.86	1.16	0.62	1.20	0.29
ΔMaximum	0.62	1.13	0.82	0.92	0.30	0.34	0.127	0.825	0.808	0.61	1.03	0.63	1.22	0.45
Initial recovery														
ΔMean ^6^	−0.45	−0.79	−0.55	−0.69	0.18	0.20	0.072	0.510	0.119	−0.53 ^ab^	−0.57 ^ab^	−0.37 ^b^	−1.02 ^a^	0.27
ΔMaximum	−0.86	−1.00	−1.03	−0.82	0.23	0.25	0.382	0.589	0.418	−0.85	−1.22	−0.87	−0.77	0.35

^1^ Main effects: Can = canola oil, Bet = betaine; ^2^ SEDm = standard error of the difference between main effects; ^3^ Treatment diet effects: BASE = basal diet, CAN = BASE plus canola oil, BET = BASE plus betaine, CB = BASE plus canola oil and betaine; ^4^ SEDt = standard error of the difference between treatments; ^5^ Δ variable = (heat challenge day 2 variable—Pre-challenge variable); ^6^ Δ variable = (Recovery day 2 variable—Recovery day 1 variable); ^a,b^ Treatment means with different superscripts are different (*p* < 0.05).

## Data Availability

The datasets used and/or analyzed during the current study are available from the corresponding author on reasonable request.
